# Evaluating the Cauchy combination test for count data

**DOI:** 10.1371/journal.pone.0334663

**Published:** 2025-10-24

**Authors:** Huda Alsulami, Silvia Liverani

**Affiliations:** 1 School of Mathematical Sciences/Centre for Probability, Statistics and Data Science, Queen Mary University of London, London, England, United Kingdom; 2 Department of Statistics, King Abdulaziz University, Jeddah, Makkah, Saudi Arabia; Tongji University, CHINA

## Abstract

The Cauchy combination test (CCT) is a *p*-value combination method used in multiple-hypothesis testing and is robust under dependence structures. This study aims to evaluate the CCT for independent and correlated count data where the individual *p*-values are derived from tests based on normal approximation to the negative binomial distribution. The correlated count data are modelled via copula methods. The CCT performance is evaluated in a simulation study to assess the type 1 error rate and the statistical power, and compare it with existing methods. Our results indicate that the number of combined tests, the negative binomial success parameter, and sample size significantly affect the type 1 error rate of the CCT under independence or moderate correlation. The CCT has more control over managing the type 1 error rate as the strength increases in the Gumbel-Hougaard copula. In general, the choice of copula and the strength of its correlation have a significant influence on type 1 error rates for both the CCT and MinP tests. Our simulation findings support the broader applications of the CCT under multivariate copulas that model upper-tail dependence with higher correlations. This knowledge may have significant implications for practical applications.

## Introduction

Combining *p*-values from various statistical tests is a fundamental procedure in multiple testing for applied statistics. It is a tool to detect an overall effect, such as in meta-analysis and bioinformatics. These combination tests combine and unify large numbers of *p*-values to a single *p*-value, potentially providing a more powerful test than testing each *p*-value separately. Suppose there are *m* hypotheses to be tested simultaneously. Let *H*_0*i*_ and *H*_*ai*_ represent the null and alternative hypotheses for the *ith* variable, respectively, where i=1,…,m. Let $T={\left(T_{1},...,\;T_{m}\right)}^{T}$ be the vector of test statistics corresponding to the *m* hypotheses, along with their associated *p*-values $P\;=\;{\left(p_{1},...\;,\;p_{m}\right)}^{T}$. The purpose of the *p*-value combination test is testing:

$$H_{0}:\overset{m}{\underset{i=1}{⋂}}H_{0i}\;\text{versus}\;H_{a}:\overset{m}{\underset{i=1}{⋃}}H_{ai}$$ (1)

where *H*_0_, the global null hypothesis, is satisfied when all the individual null hypotheses *H*_0*i*_ are true and *H*_*a*_, the global alternative hypothesis, if at least one of the individual alternatives *H*_*ai*_ is true. Combining multiple tests provides a comprehensive conclusion about a specific research question. Moreover, it improves statistical power and controls the inflation of type 1 errors. These *p*-value combination methods inherently account for the number of combined tests, thereby avoiding the need for multiple testing corrections.

In the literature, many *p*-value combination tests, which differ by their underlying assumptions, have been proposed to combine independent individual *p*-values [1,2]. Extensions of these combination tests have been developed to include dependence and weights [3–7]. The significance of the global alternative hypothesis may be significantly affected when the test statistic of the combined *p*-values does not appropriately account for the correlations among the individual *p*-values. It is crucial to investigate the impact of the correlation on the significance of the combined test [8]. For an overview of these methods see [8–11]. Recently, there has been interest in the Cauchy combination test (CCT) [12] due to its advantageous features over other methods in addressing challenges arising from correlations, computations, and sparse signals in high-dimensional settings.

This study set out to evaluate the CCT’s performance for count data. The objective is to investigate the type 1 error rate and the statistical power of the CCT when the individual *p*-values are derived from test statistics based on the normal approximation to the negative binomial distribution. The study offers some important insights by studying the influence of the negative binomial parameter, the success parameter, and the number of individual *p*-values on the combination test. Moreover, it discusses the implications on the power in the case of independent and correlated data.

This paper is organized as follows. The next section, Materials and methods, reviews three *p*-value combination methods: Fisher, MinP, and the Cauchy combination tests, and introduces the copula methods to construct correlations between count variables. The Simulation study section describes the simulation design. The Results and discussion section presents findings and applications to meta-analysis data. Finally, a conclusion is given in the Conclusion section.

## Materials and methods

In this section, we briefly review the CCT and two other *p*-value combination tests, the MinP and Fisher’s tests. In addition, we introduce the copula methods for producing correlated data.

### *P*-value combination methods

#### MinP test.

The minimum *p*-value test (MinP) [13], orders the individual *p*-values in ascending order $p_{\left(1\right)}<p_{\left(2\right)}<\ldots <p_{\left(m\right)}$. Under the assumption that they are independent and identically uniform on the unit interval [0,1], the MinP test is *p*_(1)_ which follows a beta distribution with parameters 1 and *m*, and its *p*-value is calculated as $1-{\left(1-p_{\left(1\right)}\right)}^{m}$.

#### Fisher’s combination test.

The Fisher’s combination test [1] combines the non-linear transformations of the *m p*-values where each transformation $-2\log(p_{i})$, under the null hypothesis, has a chi-square distribution with 2 degrees of freedom. Therefore, the Fisher’s test statistic has the following distribution:

$$\Psi_{F}=\sum_{i=1}^{m}-2\log(p_{i})\sim \chi_{2m}^{2}.$$ (2)

#### Cauchy combination test.

The Cauchy combination test (CCT) by Liu and Xie [12] possesses advantageous features over other tests. The CCT is robust against correlation structure and powerful against sparse signals. In addition, it is computationally efficient which makes it suitable for high dimensional data analysis. The CCT is the sum of weighted non-linear *p*-values transformations through the tangent function. Under the null hypothesis, the CCT is defined as:

$$\Psi_{CCT}=\sum_{i=1}^{m}\omega_{i}\tan\{(0.5-p_{i})\pi \},$$ (3)

where $\omega_{i}>0$ and $\sum_{i=1}^{m}\omega_{i}=1$. If no prior information is provided, the CCT becomes the weighted average of the transformations where $\omega_{i}=\frac{1}{m}$. However, if the weights are random variables and independent of the individual test statistics, then the tail approximation still holds. Under various correlation structures, the correlation has a minimal impact on the tails of the CCT distribution. The tails of the CCT distribution are approximately standard Cauchy, and its *p*-value is:

$$P_{CCT}=0.5-\frac{\arctan(\psi_{CCT})}{\pi }.$$ (4)

It has been theoretically and empirically demonstrated in [12] that the CCT can effectively control type 1 error rates across different significance levels. The ratio of the size of the CCT, which represents the type 1 error rate, to the significance level approaches 1 as the significance level converges to 0. This indicates its validity in large-scale multiple testing.

Under the global null hypothesis, the tail probability of the CCT is approximated by a standard Cauchy distribution, which is valid under the assumptions of bivariate normality of the individual tests and some regularity conditions on the correlation matrix. However, Long et al. [14] broaden the applicability of the CCT when the assumption of bivariate normality may not hold. They demonstrated that, theoretically and by simulation, the approximation of the standard Cauchy distribution for the tail probability of the CCT is still valid across a broader range of bivariate copula distributions. This includes the six popular copula distributions, the product Copula, Farlie–Gumbel–Morgenstern (FGM) Copula, Cuadras-Augé Copula, Normal Copula, Ali-Mikhail-Haq (AMH) Copula, and Survival Copula. While their study focused on bivariate copula dependence structures, our research is based on simulating more complex joint distributions under multivariate copulas, which is realistic for many real-world applications where higher-order correlations exist.

### Copula methods

We utilise copulas to simulate correlated count data [15,16]. Copula methods are powerful tools to capture complex dependencies between variables rather than simple linear relationships [17]. They model various structures of dependencies, including tails dependencies. A copula is a multivariate distribution function that models the dependence structure between multiple variables, each following a standard uniform marginal distribution, U(0,1) [18]. The basic theorem of copula theory is known as Sklar’s Theorem [19]. Two types of Archimedean parametric copulas for asymmetric dependencies are considered: the Clayton and the Gumbel-Hougaard copulas. As both copulas are widely used in applications to model asymmetric tail dependence, they are suitable for evaluating the CCT and studying the effect of low and large correlated *p*-values on the properties of the combination tests. In addition, they are exchangeable copulas as their bivariate margins share a common correlation structure through Kendall’s tau value. They are easy to interpret and serve our aim to evaluate the combination method under a simple controlled correlation structure; for example, if the original data exhibit jointly low counts using the Clayton copula.

The Clayton copula models positive dependence in the lower tail, while the Gumbel-Hougaard copula models positive dependence in the upper tail. They are defined by:

$$C(u;\theta )={\left(1-m+\sum_{i=1}^{m}u_{i}^{-\theta }\right)}^{-\frac{1}{\theta }},\;\theta >0,\;u\in {\left[0,1\right]}^{m}$$ (5)

and

$$C(u;\theta )=\exp\left\{-{\left[\sum_{i=1}^{m}(-\log u_{i}{)}^{\theta }\right]}^{1/\theta }\right\},\;\theta \in [1,\infty ),\;u\in {\left[0,1\right]}^{m}.$$ (6)

Both copulas have the parameter *θ*, representing the tail dependence coefficient. As *θ* increases, the strength of dependence increases. The associated Kendall’s *τ* for each copula depends on the parameter *θ*. It takes the form τ=θ/(θ+2) for the Clayton copula and τ=1−1/θ for the Gumbel-Hougaard copula[18]. For illustration, S1 Fig and S2 Fig present wireframe and contour plots of bivariate Clayton and Gumbel–Hougaard copulas, and scatter plots of a sample of size n = 1000 simulated from the bivariate copulas with τ=0.5 (θ=2).

### Evaluating the Cauchy combination test for count data

Biological data, such as RNA-sequencing data, are best fitted with the negative binomial distribution. Unlike the Poisson distribution, the negative binomial distribution accounts for overdispersion when the variance exceeds the mean [20,21]. In this context, the CCT has been adopted as a gene-set test to combine *p*-values from individual genes and identify differentially expressed genes [22]. Another application of the CCT on count data, in a comparative study evaluating different methods for analysing microbiome data [23], the CCT outperforms other methods of combining *p*-values and provides an accurate *p*-value while controlling the type one error rate. In addition, the ranked combined *p*-values produced from the CCT have high-rank similarity with the true ranks. The CCT successfully replicated and identified microbiome taxa associated with colorectal cancer in a real dataset where the most highly ranked microbiome taxa using the CCT have been reported to be associated with this condition. Consequently, evaluating the CCT is pivotal to providing a robust statistical tool for analyzing non-Gaussian count data.

We aim to evaluate the type 1 error rate and the power of the CCT to combine individual *p*-values obtained from the normal approximation to the negative binomial distribution and modelling the correlation between the discrete data via copula methods. There are several formulations for the negative binomial distribution in the literature. In this paper, we used the following definition. In a sequence of independent Bernoulli trials, the negative binomial distribution is the distribution of the number of trials (or failures) *X* needed until a fixed number (*r*) of successes occurs. Then, X~NB(r,p) where the first parameter *r* is the number of successes, and *p* is the probability of success in each trial, and the probability mass function of *X* is:

f(x)=(x+r−1r−1)pr(1−p)x,x=0,1,... (7)

with mean and variance:

$$E(X)=\frac{r(1-p)}{p}\;\text{and}\;V(X)=\frac{r(1-p)}{p^{2}}.$$ (8)

The normal approximation to the negative binomial is applied here for large *r* and moderate *p*. This approximation is accurate under these conditions because both parameters, *r* and *p*, affect the shape and symmetry of the negative binomial distribution. Hence, it closely resembles that of a normal distribution. This approximation enables us to meet the assumptions required by the Cauchy combination test for the individual tests. The normal approximation to the negative binomial distribution becomes as follows:

X≈N(E(X),V(X)). (9)

Then, we approximate the sampling distribution of the sample mean using the central limit theorem where the sampling distribution converges to normal for large sample size.

## Simulation study

This simulation was designed to assess the type 1 error rates and the power of three different *p*-value combination methods: the Cauchy combination test (CCT), Fisher’s test, and the MinP test using independent and correlated *p*-values obtained from the normal approximation to the negative binomial distribution. In this context, the number of variables refers to the number of individual tests, or similarly *p*-values, denoted as *m*. We denote the sample size as *n* and the number of simulations as *M*.

### Data generation

Datasets for independent and correlated variables were simulated from the negative binomial (NB) distribution using the R software version 4.5.0 [24]. The correlations were modelled using the Clayton and the Gumbel-Hougaard copulas to introduce the correlation among the variables using the copula package [17]. The dependence parameter, *θ*, denotes the dependency strength between the variables. We considered *θ* values of 1, 3, and 5, which represent weak (independence in the Gumbel-Hougaard copula), moderate, and strong correlations in the lower or upper tail. The corresponding Kendall’s *τ* values are 0.33, 0.60, and 0.71 for the Clayton copula, and 0, 0.67, and 0.80 for the Gumbel-Hougaard copula. First, we generated data using copulas with dimension *m* and parameter *θ*. Following this, the simulated unit variates from these copulas were transformed by the negative binomial quantile function to obtain count data from the negative binomial distribution.

### Type 1 error rate

To evaluate the type 1 error rate, the data were simulated under the null hypothesis, *H*_0*i*_: $\mu_{i}=\mu_{0i}$, i=1,…m, using *M* = 10^5^ replications at 0.05 and 0.01 levels of significance. We generated datasets from negative binomial distribution with parameters *r* and *p* = 0.5, *NB*(*r*,0.5), each with a sample size *n* = 30 under the null hypothesis *H*_0*i*_: $\mu_{i}=r_{0i}$, i=1,…m. Fixing a large sample size of 30 and a moderate probability of success of 0.5, helped us to isolate their effects and satisfy part of the assumptions of the normal approximation and the CLT, and then, study the influence of the success parameter *r*. We varied the following parameters: the number of variables *m*, the number of success parameter of the negative binomial distribution *r*, and copula parameter *θ*. We calculated the Z test for each variable as $Z_{i}=\frac{\bar{X_{i}}\;-\;\mu_{0i}}{\sqrt{\frac{V(X_{i})}{n}}}$, with a two-sided *p*-value given by $2[1\;-\;\Phi (|Z_{i}|)$, for i=1,…m. We combined the individual *p*-values using the three combination methods. Finally, we calculated the mean of the combined *p*-values that were below the prespecified significance level.

### Power comparisons

The statistical powers of the three combination methods were compared in the presence of sparse signals. The evaluation was performed against the sample size and the correlation strength using different correlation structures. Data were generated from a negative binomial distribution consisting of nine variables with parameters *r* = 10 and *p* = 0.5, along with one variable with *r* = 11 and *p* = 0.5. This scenario reflects the sparsity of the signals commonly encountered in multiple hypothesis testing, where a small number of hypotheses compared to the nulls are true. Specifically, in our simulation setting, a significant test out of 10 is an example of this scenario. In practice, for instance, detecting rare variants in the Genome-Wide Association Studies (GWAS) or the RNA-seq analysis, few genes are expected to be associated with a phenotype or a disease.

We varied the correlation structures and evaluated the power against values of sample size n=5,10,30,50,100,150,200,300,500,1000 using M=10,000 replicated samples. The correlation structures included independence among all variables, and correlated variables modelled via the Clayton and the Gumbel-Hougaard copulas with *θ* equals 3.

## Results and discussion

To evaluate the performance of the CCT, we conducted a simulation study to assess the type 1 error and power when the individual *p*-values were obtained from tests based on the normal approximation to the negative binomial distribution. In addition, we studied the effect of the success parameter, the sample size, and the correlation structures. In addition, we provide applications of the combination methods to real meta-analysis datasets for count data.

**Fig 1:**
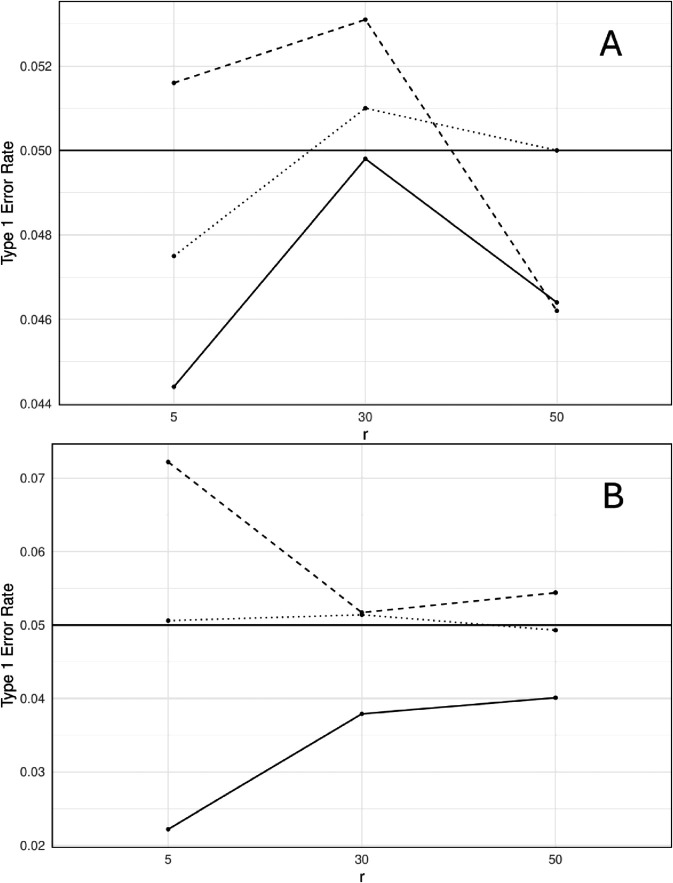
Type 1 error rates of the Cauchy combination test (smooth line), Fisher’s test (dotted line), and MinP test (dashed line) at the significance level α = 0.05 against the success parameter r, using M = 10,000 replications. Datasets were simulated from *m* independent negative binomial variables with sample size *n* = 30 and parameters (*r*,0.5). A: *m* = 10. B: *m* = 50.

Table 1 presents the type 1 error rates for different numbers of independent *p*-values, *r*, and significance levels. To demonstrate the effect of the success parameter *r*, Fig 1 presents the results when the numbers of combined tests are 10 and 50. Across different values of the parameter *r* and varying the number of tests *m*, the Fisher’s test consistently controls the type 1 error well. When the number of tests is small, all methods manage to control false positives within different significance thresholds. However, the impact of the parameter *r* on the type 1 error rates of the CCT and MinP tests is evident as *m* increases.

**Table 1:** Type 1 error rates of the CCT, Fisher, and MinP tests at different values of the level of significance α using *M* = 10,000 replications. Datasets were simulated from *m* independent negative binomial variables NB(*r*, 0.5) with sample size *n* = 30, where *r* is the success parameter and the probability of success is 0.5.

Number of tests (*m*)	*r*	Test	α	
			0.05	0.01
10	5	CCT	0.0444	0.0106
		Fisher	0.0475	0.0097
		MinP	0.0516	0.0142
	30	CCT	0.0498	0.0086
		Fisher	0.0510	0.0103
		MinP	0.0531	0.0096
	50	CCT	0.0464	0.0100
		Fisher	0.0500	0.0120
		MinP	0.0462	0.0095
50	5	CCT	0.0222	0.0058
		Fisher	0.0506	0.0110
		MinP	0.0722	0.0188
	30	CCT	0.0379	0.0066
		Fisher	0.0514	0.0086
		MinP	0.0517	0.0109
	50	CCT	0.0401	0.0085
		Fisher	0.0493	0.0096
		MinP	0.0544	0.0112
100	5	CCT	0.0078	0.0019
		Fisher	0.0495	0.0091
		MinP	0.0665	0.0198
	30	CCT	0.0230	0.0034
		Fisher	0.0526	0.0113
		MinP	0.0494	0.0097
	50	CCT	0.0259	0.0061
		Fisher	0.0469	0.0085
		MinP	0.0484	0.0098

CCT: Cauchy combination test; MinP: Minimum *P*-value test.

The MinP method exhibited relatively stable type 1 error rates as *r* increased while it remained conservative for the CCT. The CCT had conservative type 1 error rates at the 0.05 significance level, whereas at 0.01, it was around 0.01, except when *m* = 100 and *r* equals 5 or 30. As *r* increased, the rate increased for the CCT especially for large *m*. For instance, when *m* = 50 at α=0.05, type 1 error rate increased from 0.0222 to 0.0401.

The results in Table 1 show that a small value of *r* (*r* = 5) leads to conservative type 1 error rates for the CCT and liberal rates for the MinP method. We expect that as the parameter *r* value increases, the rate will be around the significance level of 0.05. A possible interpretation is that the skewness of the negative binomial distribution affects the sampling distribution of the sample mean. When *r* is small, the negative binomial distribution is positively skewed, and its skewness is defined as $\gamma =\frac{\left(2-p\right)}{\sqrt{(1-p)r}}$. The skewness value decreases and becomes closer to zero as *r* increases, which affects the symmetry of the sampling distribution and, therefore, the accuracy of the individual *p*-values.

When *r* is small, the normal approximation of the sample mean through the Central Limit Theorem might be inaccurate and produce *p*-values, under the null hypothesis, that are stochastically larger than a uniform distribution U(0,1) (conservative individual *p*-values). As a result, the MinP method may still have higher rejection rates because of its sensitivity to the few small *p*-values. The CCT, which is based on the average of the transformed *p*-values, is less likely to reject the null hypothesis due to the excess number of large *p*-values. In practical contexts, the trade-off between type 1 error rate and power is crucial. Applying the CCT or MinP method has implications on the results that rely on the normal approximations to skewed count data, such as negative binomial data with small *r*. The CCT has a conservative type 1 error rate and reduces false positives, i.e. rejecting the null hypotheses less frequently than the expected nominal level. Consequently, it has less power and may fail to detect true positives (true signals). On the other hand, the MinP method increases the risk of false positives, and therefore, has higher misleading power. For example, in applications such as genomics, the aim is to detect differentially expressed genes; The MinP method may incorrectly detect genes that are not truly differentially expressed, and the CCT may not be able to declare the truly significant genes.

In contrast, the Fisher method shows stable type 1 error rates closer to the nominal level regardless of the value of the success parameter *r*. This indicates its robustness to the value of *r* and means that even when we have conservative individual *p*-values, Fisher tends to control the type 1 error rate. This finding is also supported by [25], who found that the Fisher method works well with conservative null *p*-values.

As a practical guideline, the success parameter *r* should be sufficiently large. A lower bound on *r* should be maintained such that the standardised skewness (the skewness of the sample mean), $\frac{\gamma }{\sqrt{n}}$, is below a small threshold that is close to zero. A sensitivity analysis of the CCT type 1 error rate was conducted by varying the success parameter *r*, the sample sizes n=30,100,200, and the number of combined tests m=30,50. The results are presented in Table 2. For example, when *m* = 30, and across different values of sample sizes 30,100,200, we observed that the CCT type 1 error rates were around 0.05 (after rounding to two digits) when the standardised skewness values ranged between 0.05 and 0.07. A smaller sample size requires a larger *r* value, and conversely, a larger sample size requires a smaller *r*. Specifically, when the sample sizes *n* were 30,100 or 200, the corresponding *r* values that maintain the type 1 error rate were 50,20 and 5, respectively. Additionally, a larger number of individual tests required a more stringent threshold. For instance, when *m* = 50, (the results are not shown), the thresholds were around 0.04–0.05. This approach may improve the normal approximation of the individual test statistics, and therefore, the individual *p*-values follow the uniform distribution under the null. It helps to effectively control the type 1 error rate of the combination methods.

**Table 2:** Sensitivity analysis of the type 1 error rates of the CCT using *M* = 10,000 replications. Datasets were simulated from *m* = 30 independent negative binomial variables NB(*r*, 0.5) with varying sample sizes, where *r* is the success parameter and the probability of success is 0.5.

r	n=30		n=100		n=200	
	sk	CCT	sk	CCT	sk	CCT
5	0.17	0.036	0.09	0.043	0.07	0.045
10	0.12	0.038	0.07	0.043	0.05	0.046
15	0.10	0.042	0.05	0.043	0.04	0.049
20	0.09	0.041	0.05	0.047	0.03	0.047
25	0.08	0.044	0.04	0.046	0.03	0.048
30	0.07	0.042	0.04	0.045	0.03	0.049
35	0.07	0.042	0.04	0.047	0.03	0.049
40	0.06	0.044	0.03	0.046	0.02	0.047
45	0.06	0.043	0.03	0.046	0.02	0.050
50	0.05	0.050	0.03	0.045	0.02	0.047
55	0.05	0.048	0.03	0.048	0.02	0.049
60	0.05	0.044	0.03	0.049	0.02	0.052
65	0.05	0.048	0.03	0.048	0.02	0.047
70	0.05	0.046	0.03	0.048	0.02	0.050
75	0.04	0.046	0.02	0.050	0.02	0.050
80	0.04	0.046	0.02	0.045	0.02	0.050
85	0.04	0.044	0.02	0.046	0.02	0.047
90	0.04	0.047	0.02	0.047	0.02	0.052
95	0.04	0.048	0.02	0.048	0.02	0.048
100	0.04	0.044	0.02	0.048	0.02	0.052

CCT: Cauchy combination test; sk: Standardised skewness.

**Fig 2:**
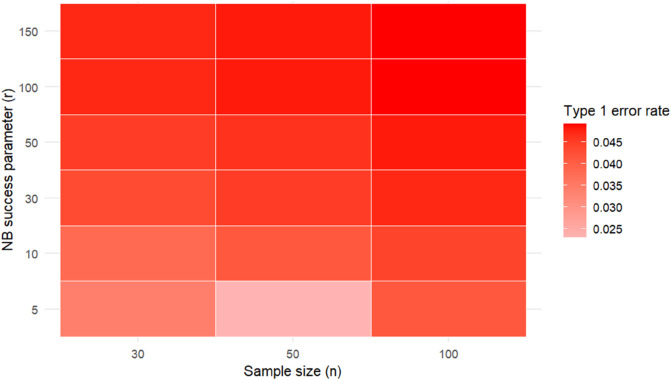
A heatmap presenting the average type 1 error rates of the Cauchy combination test at the significance level α = 0.05.

In addition, we conducted a rigorous diagnostic assessment using a heatmap in Fig 2 of the CCT type 1 error rates across a grid of different sample sizes *n* and success parameters *r*. In 1000 replications, we assessed the average of type 1 error rates using 10,000 simulations by combining *m* = 30 individual *p*-values. The heatmap shows that as *n* and *r* increase, type 1 error rates approach the nominal level of 0.05.

It is important to note that the choice of sample size of 30 is based solely on the normal approximation using the CLT. Although it is considered large enough, in practical applications, there are more rigorous methods to estimate the sample size and evaluate the precision of the normal approximation of skewed count data; see, for example, [26]. This may help explain the observed conservative type 1 error rates for large numbers of individual tests (*m*) and (*r*), meaning that the CCT requires larger sample sizes. As both the negative binomial success parameter (*r*) and sample size (*n*) increase, the distribution of the sample mean becomes more symmetric and continuous, and the corresponding *p*-values better approximate the uniform distribution under the null. Consequently, the CCT rejection rate stabilizes around the nominal level, confirming the theoretical validity of the method under approximately continuous individual *p*-values. With lower values of (*r*) and (*n*), the individual tests are influenced by the discreteness and skewness of the underlying count data and produce discrete *p*-values that may affect the validity of the CCT. Alternatively, the Fisher method is more appropriate than other combination methods under the assumption of independence.

Two types of correlation structures using the Clayton and the Gumbel-Hougaard copulas were introduced. The results are presented in Tables 3 and 4. Fisher’s test has the highest type 1 error rates, except in the Gumbel-Hougaard copula when *θ* is 1, which represents the independence case. As expected, this is due to the violation of the independence assumption. In contrast, the MinP test tends to be more conservative in controlling type 1 errors across different copula structures and levels of dependence. Our findings show that the CCT outperforms the MinP test in controlling false positives, particularly when the dependence strength *θ* increases from 1 to 5 in the Gumbel-Hougaard copula.

**Table 3:** Type 1 error rates for the CCT, Fisher, and MinP tests at 0.05 significance level using *M* = 10,000 replications. Datasets were modeled using the Clayton copula with θ={1,3,5} and simulated from *m* negative binomial variables NB(*r*, 0.5) with sample size *n* = 30, where *r* is the success parameter and the probability of success is 0.5.

Number of tests (*m*)	*r*	Test	θ		
			1	3	5
10	5	CCT	0.0557	0.0604	0.059
		Fisher	0.106	0.156	0.179
		MinP	0.0489	0.0346	0.0294
	30	CCT	0.0562	0.0603	0.0588
		Fisher	0.1193	0.1679	0.1864
		MinP	0.0418	0.0286	0.0251
50	5	CCT	0.0476	0.067	0.066
		Fisher	0.1841	0.2399	0.2574
		MinP	0.0517	0.0317	0.0261
	30	CCT	0.0624	0.0665	0.06
		Fisher	0.2016	0.2548	0.2648
		MinP	0.0359	0.0196	0.0141
100	5	CCT	0.0351	0.0713	0.0705
		Fisher	0.221	0.2689	0.2834
		MinP	0.0493	0.0311	0.0208
	30	CCT	0.0594	0.0691	0.0625
		Fisher	0.2353	0.281	0.2908
		MinP	0.0376	0.0201	0.012
500	5	CCT	0.0057	0.049	0.0654
		Fisher	0.28	0.2948	0.3103
		MinP	0.0611	0.0288	0.0194
	30	CCT	0.03	0.065	0.0595
		Fisher	0.29	0.3031	0.3192
		MinP	0.0355	0.0155	0.0081

CCT: Cauchy combination test; MinP: Minimum *P*-value test.

**Table 4:** Type 1 error rates for the CCT, Fisher, and MinP tests at 0.05 significance level using *M* = 10,000 replications. Datasets were modeled using the Gumbel-Hougaard copula with θ={1,3,5} and simulated from *m* negative binomial variables NB(*r*, 0.5) with sample size *n* = 30, where *r* is the success parameter and the probability of success is 0.5.

Number of tests (*m*)	*r*	Test	θ		
			1	3	5
10	5	CCT	0.0478	0.0529	0.0498
		Fisher	0.0522	0.1932	0.2025
		MinP	0.0534	0.0196	0.0138
	30	CCT	0.0492	0.0539	0.051
		Fisher	0.0533	0.1918	0.2014
		MinP	0.0527	0.0193	0.0131
50	5	CCT	0.0233	0.0536	0.0506
		Fisher	0.0521	0.2707	0.2827
		MinP	0.0669	0.0092	0.0053
	30	CCT	0.0402	0.0568	0.0514
		Fisher	0.0513	0.2722	0.2825
		MinP	0.0528	0.0086	0.0044
100	5	CCT	0.0104	0.0542	0.051
		Fisher	0.0507	0.2948	0.3051
		MinP	0.0633	0.0057	0.003
	30	CCT	0.0236	0.0559	0.0511
		Fisher	0.0521	0.2937	0.3046
		MinP	0.0484	0.0061	0.0022
500	5	CCT	0.0001	0.0554	0.0505
		Fisher	0.0469	0.3255	0.3342
		MinP	0.0868	0.0036	0.0014
	30	CCT	0.0004	0.0555	0.0512
		Fisher	0.0478	0.3259	0.3351
		MinP	0.0557	0.0032	0.0011

CCT: Cauchy combination test; MinP: Minimum *P*-value test.

For the CCT, the choice of copula model significantly affected controlling the type 1 error rates. In Table 3, as the parameters *r* and *θ* increased, the CCT had slightly higher type 1 error rates for different numbers of dependent tests through the Clayton copula. On the other hand, modelling tests based on the Gumbel-Hougaard copula showed that as the correlation strength increased, the type 1 error rate decreased and became more controlled for highly correlated tests. For example, at *r* = 30 and θ=5, across different numbers of tests, the type 1 error rates for the CCT ranged from 0.0588 to 0.0625, while in the Gumbel-Hougaard copula they ranged from 0.051 to 0.0514. Similarly, when *r* = 30 and θ=3, CCT, the type I error rates ranged from 0.0691-0.0603, but in the Gumbel-Hougaard copula, they were between 0.0539-0.0568.

Both copulas differ in modelling extreme values. The dependence in the lower tail of the Clayton copula tends to produce small counts that occur together across variables, resulting in low sample means and large absolute Z test statistics values. Furthermore, the discreteness of the data can lead to tied sample means and *p*-values. When these effects are combined, small *p*-values can occur more frequently than expected under the global null hypothesis. Since the CCT is sensitive to small *p*-values, this can significantly inflate the type 1 error rate. On the other hand, the Gumbel-Hougaard copula better controls type 1 error rates because it shows weak dependence in the lower tail. Thus, the sample means increase compared to Clayton and therefore reduce the likelihood of many false positives under the null hypothesis.

Under multivariate copulas, lower-tail dependence structure can inflate the type 1 error rate, as shown in the Clayton copula results, and copulas that model the upper tail are valid for higher correlations, i.e. higher copula parameter (θ). We further explored the validity of the CCT under the multivariate survival Clayton copula [27]. Similar to the Gumbel-Hougaard copula, this copula captures upper-tail dependence. We aimed to assess whether the tail dependence structure affects the type 1 error rate of the CCT. It was proved that under the bivariate survival copula, the CCT is valid and its tail is well approximated by the Cauchy distribution [14]. When considering the multivariate copula the results, see S1 Table, indicated that the CCT maintained type 1 error rates under the survival Clayton copula for higher copula parameter θ={5,8}. These findings support the broader applications of the CCT to multivariate copulas to model the upper tail dependence.

**Fig 3:**
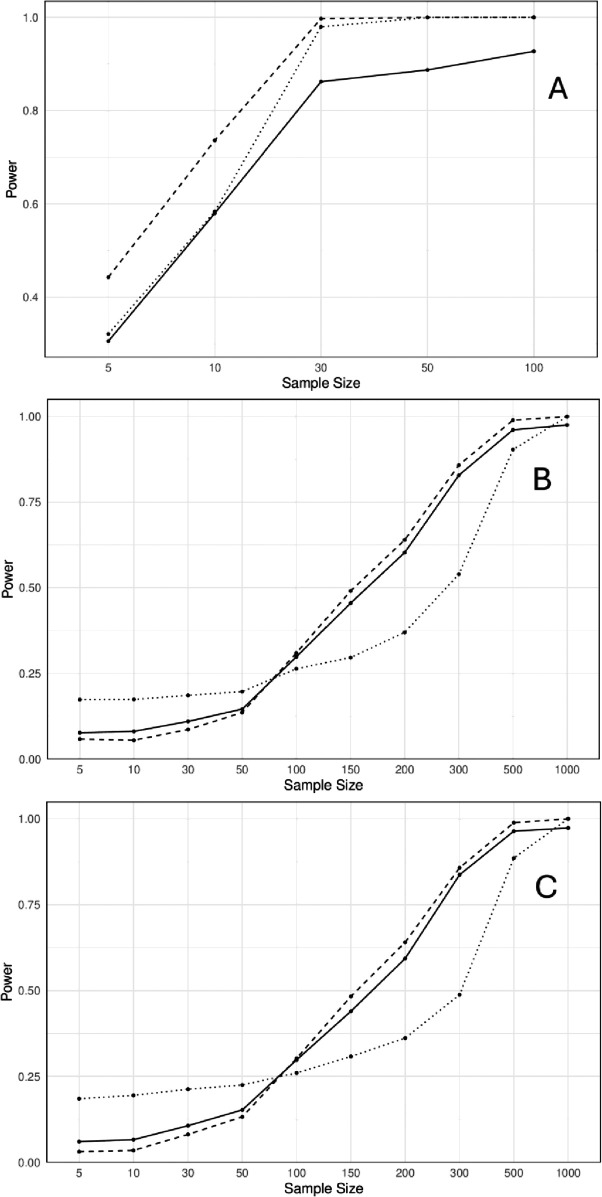
The power comparison of the Cauchy combination test (smooth line), Fisher’s test (dotted line), and MinP test (dashed line) from negative binomial data in three different correlation structures. A: Independent variables. B: Clayton copula. C: Gumbel-Hougaard copula.

Fig 3 compares the power of the three combination methods against the sample size. As expected, power is generally increasing as the sample size increases. The Fisher and MinP tests have higher power than the CCT when the combined individual tests are independent. The CCT and MinP exhibit comparable power in sparse signals and small effect sizes, regardless of the correlation structure.

The MinP method showed relatively higher power compared to the Fisher method and the CCT when combining independent tests. At a sample size of 30, both the MinP and Fisher methods approached the maximum power of 1, while the power curve for the CCT increased slowly. It remained below the other two methods even for larger sample sizes, which suggested that it may be conservative in detecting the true signal when *r* is small, specifically when *r* = 11, where the normal approximation may not be accurate.

For correlated count data, the Fisher method exhibited higher power, especially for small to moderate sample sizes. The independence assumption for the Fisher method was violated in this case, leading to inflated type 1 error rate, which reflected a misleading increase in power. Although the power curve for the CCT achieved higher power at smaller sample sizes, this must be interpreted with caution due to its liberal type 1 error rates at *n* = 30. However, as the sample size exceeded 30, the normal approximation of the negative binomial variables became more accurate, and the power observed at larger sample sizes became more reliable. On the other hand, the MinP method, which represents a conservative method under dependence, offered more reliable power results.

### Real data applications

In addition to simulation results, we provide a real data analysis to illustrate the application of combination methods to combine *p*-values from count data. Meta-analyses of Genome-Wide Association Studies (GWAS) show that two SNPs (rs4570625-T and rs17110747-A) on the TPH2 gene are associated with major depressive disorder (MDD) using fixed effects models [28]. We apply the Cauchy combination test (CCT) and the Fisher method to combine *p*-values in two meta-analysis studies. The first study includes six independent case-control studies to test the association between the rs4570625-T SNP and MDD. The second meta-analysis involves five studies to test the association of the rs17110747-A SNP and MDD. The individual *p*-values were calculated from the forest plots 1 and 2 in [28].

For the first meta-analysis, the individual *p*-values were 0.78, 0.002, 0.74, 0.016, 0.89, and 0.10. By combining these *p*-values and at a significance level of 0.05, both the CCT and Fisher method indicated significant results with combined *p*-values of 0.011 and 0.009, respectively.

In the second meta-analysis, the individual *p*-values were 0.94, 0.0015, 0.97, 0.79, and 0.81. The CCT produced a combined *p*-value of 0.0082, while the Fisher method yielded a non-significant result of 0.17. Notably, [29] applied a new proposed *p*-value combination method to combine the five individual *p*-values which resulted in a combined *p*-value of 0.0081. Along with the CCT, their result was the only significant finding among other existing *p*-value combination methods.

The Cauchy combination test (CCT) yielded significant findings when applied to both real meta-analysis studies, while the Fisher method exhibited significance in only one study. Under the independence assumption, the difference in detecting true signals between the CCT and Fisher method is due to their sensitivities to small *p*-values in a sparse setting. These findings highlight the potential of the CCT when applied to meta-analysis of count data and only a subset of studies exhibit strong effects.

It is important to note that multivariate copulas with discrete marginal distributions do not have a unique copula representation [19]. The imitation of non-uniqueness in copulas arises in the estimation of the copula parameter and joint distribution of observed discrete data. In practice, a specific copula may not capture this, but different possible copulas may lead to the same marginals and joint distribution. However, this limitation does not affect the validity of our simulation study. We explicitly specified a known correlation structure in advance, simulated latent uniform variables using a copula, and then applied the inverse cumulative function of the negative binomial distribution. The goal was to assess the influence of the correlation structures on the *p*-value combination methods [15,16].

Further study is needed to evaluate combination methods, particularly the CCT, under highly dispersed count data using distributions such as the Poisson Inverse Gaussian (PIG) and Sichel. Models based on these distributions offer flexibility in fitting and modelling highly dispersed count data and outperform negative binomial models in the analysis of crash and infectious disease count data [30–32]. Furthermore, the performance of the CCT could be compared with other methods that account for dependence structures, such as the Z or Empirical Brown’s methods, in discrete settings. It is also of interest to examine the robustness of the CCT under other types of copulas such as, for instance, a symmetric and heavy-tailed dependence structure like the Student-t copula or even under more complex mixed correlation structures such as the Vine copula. Future work could evaluate the combination methods and extend them to count data that exhibit overdispersion and temporal or spatial dependence, such as traffic data, under other distributional models and more complex dependence structures [30,32]. Such comparisons would improve our understanding of the performance of *p*-value combination methods and guide practitioners in selecting appropriate approaches for real-world count data analysis.

## Conclusion

In this paper, we compare three *p*-value combination tests, where individual *p*-values are obtained from count data based on normal approximation to the negative binomial distribution. The Cauchy combination test (CCT) is a powerful and robust method against sparse alternatives under arbitrary dependence structures. The observed variations in type 1 error rates of the CCT when combining multiple independent or correlated tests based on the normal approximation emphasize the need for caution to ensure the validity of statistical inferences. We find that the number of combined tests influences the accuracy of normal approximation, which is affected by both the sample size and success parameter. In addition, the choice of the copula and its parameter are also other factors to consider. Our simulation findings support the broader application of the CCT to multivariate copulas that model upper-tail dependence with higher correlations. These factors contribute to the robustness and validity of the CCT.

## Supporting information

S1 FigGraphical representations of the bivariate Clayton copula.Wireframe plot of the bivariate Clayton copula density (top left), contour plot of the copula distribution function (top right), contour plot of the copula density (bottom left), and scatter plot of a sample of size *n* = 1000 simulated from the bivariate Clayton copula with τ=0.5 (θ=2), illustrating lower-tail dependence.(TIFF)

S2 FigGraphical representations of the bivariate Gumbel-Hougaard copula.Wireframe plot of the bivariate Gumbel-Hougaard copula density (top left), contour plot of the copula distribution function (top right), contour plot of the copula density (bottom left), and scatter plot of a sample of size *n* = 1000 simulated from the bivariate Gumbel-Hougaard copula with τ=0.5 (θ=2), illustrating upper-tail dependence.(TIFF)

S1 TableSurvival Clayton copula results.Type 1 error rates for the CCT, Fisher, and MinP tests at 0.05 significance level using M=10,000 replications. Datasets were modeled using the survival Clayton copula with θ={1,3,5,8} and simulated from *m* negative binomial variables NB(*r*, 0.5) with sample size *n* = 30, where *r* is the success parameter and the probability of success is 0.5. Abbreviations: CCT: Cauchy combination test; MinP: Minimum *P*-value test.(PDF)
