# Supplementary figures and images for: Evaluating the Cauchy combination test for count data
Source: PLoS One. 2025 Oct 24;20(10):e0334663. doi: 10.1371/journal.pone.0334663 (PMC12551897; doi:10.1371/journal.pone.0334663)

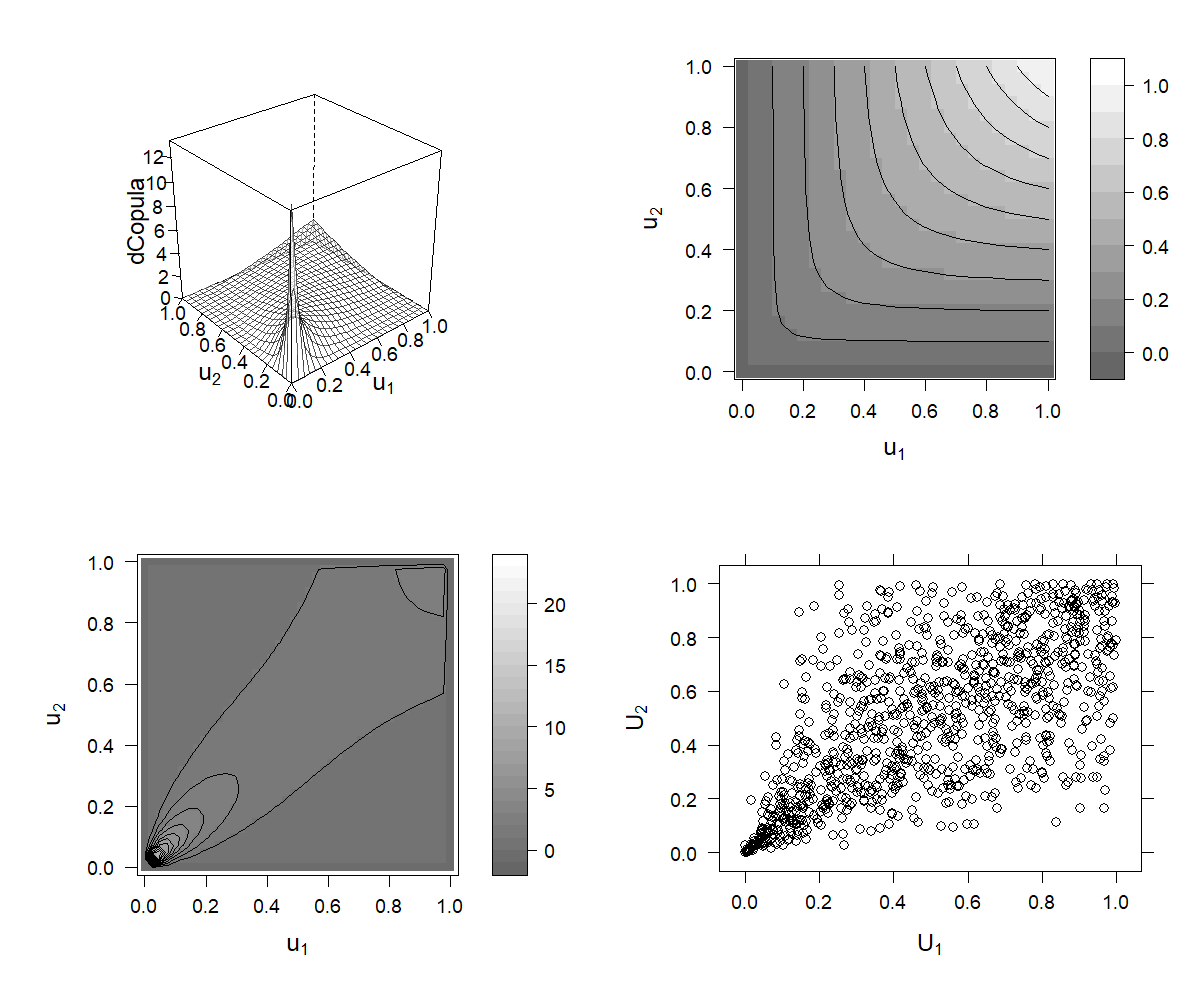

Supplement: S1 Fig — Wireframe plot of the bivariate Clayton copula density (top left), contour plot of the copula distribution function (top right), contour plot of the copula density (bottom left), and scatter plot of a sample of size n = 1000 simulated from the bivariate Clayton copula with τ=0.5 (θ=2), illustrating lower-tail dependence. (TIFF) [file pone.0334663.s001.tif]

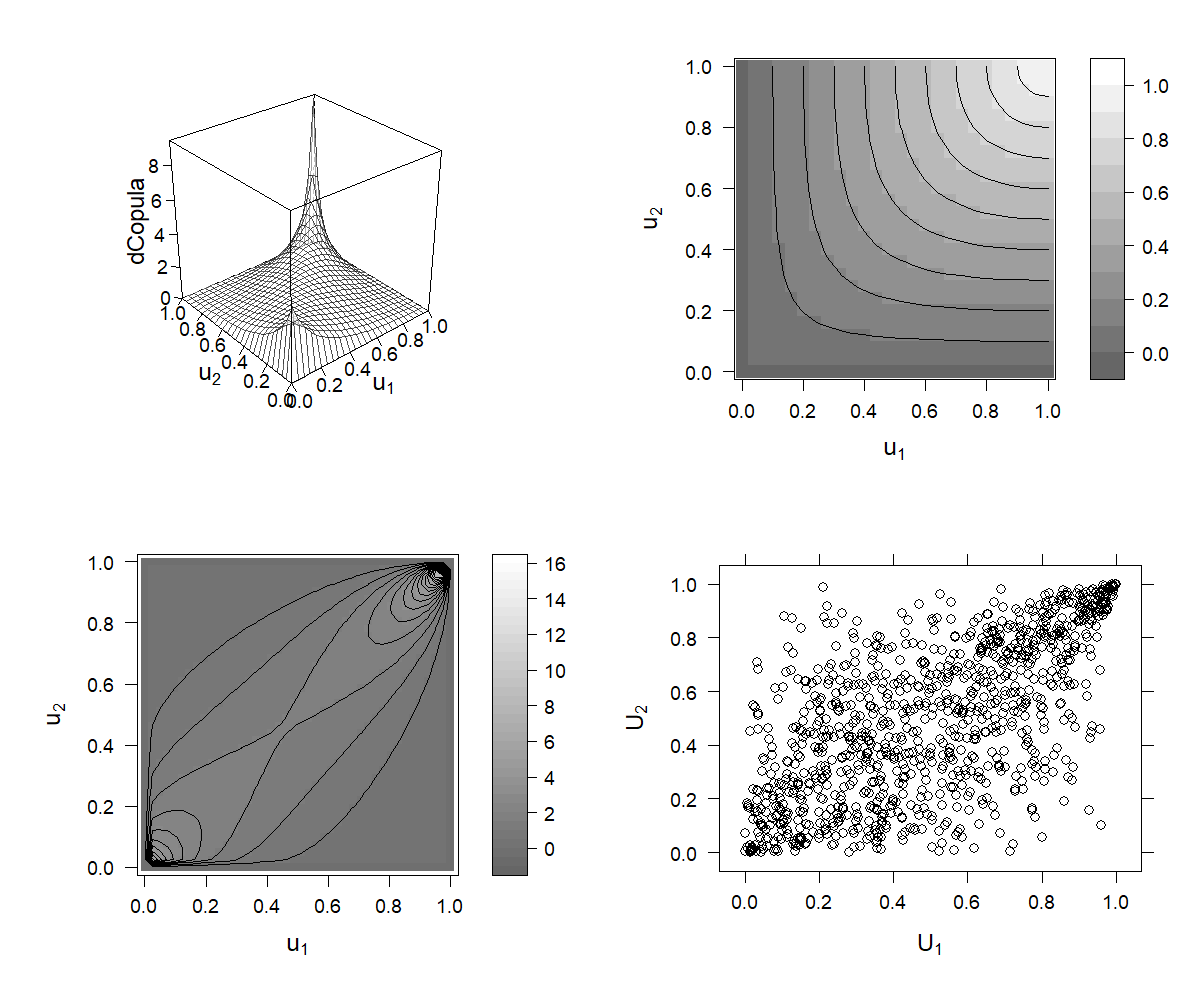

Supplement: S2 Fig — Wireframe plot of the bivariate Gumbel-Hougaard copula density (top left), contour plot of the copula distribution function (top right), contour plot of the copula density (bottom left), and scatter plot of a sample of size n = 1000 simulated from the bivariate Gumbel-Hougaard copula with τ=0.5 (θ=2), illustrating upper-tail dependence. (TIFF) [file pone.0334663.s002.tif]
