# Supplementary material for: Evaluating the Cauchy combination test for count data
Source: PLoS One. 2025 Oct 24;20(10):e0334663. doi: 10.1371/journal.pone.0334663 (PMC12551897; doi:10.1371/journal.pone.0334663)
Supplement: S1 Table — Type 1 error rates for the CCT, Fisher, and MinP tests at 0.05 significance level using M=10,000 replications. Datasets were modeled using the survival Clayton copula with θ={1,3,5,8} and simulated from m negative binomial variables NB(r, 0.5) with sample size n = 30, where r is the success parameter and the probability of success is 0.5. Abbreviations: CCT: Cauchy combination test; MinP: Minimum P-value test. (PDF) [file pone.0334663.s003.pdf]

			θ			
**m**	**r**	**Test**	**1**	**3**	**5**	**8**
10	5	CCT	0.0562	0.0541	0.0539	0.0486
		Fisher	0.1383	0.1852	0.1983	0.2011
		MinP	0.035	0.022	0.0164	0.0058
	30	CCT	0.0571	0.0562	0.0548	0.0507
		Fisher	0.134	0.1804	0.1944	0.2014
		MinP	0.038	0.0238	0.0196	0.0067
50	5	CCT	0.0585	0.056	0.0507	0.0497
		Fisher	0.2195	0.2677	0.2733	0.2807
		MinP	0.0284	0.0099	0.0063	0.0044
	30	CCT	0.0625	0.0613	0.0516	0.0508
		Fisher	0.2165	0.2664	0.2707	0.2766
		MinP	0.0276	0.0115	0.0073	0.0049
100	5	CCT	0.0579	0.0555	0.0534	0.0511
		Fisher	0.2525	0.2953	0.3052	0.3044
		MinP	0.0238	0.0078	0.0047	0.001
	30	CCT	0.0642	0.0609	0.0575	0.0529
		Fisher	0.248	0.2897	0.3006	0.3053
		MinP	0.0264	0.0119	0.0058	0.0009
500	5	CCT	0.0302	0.0564	0.0531	0.0487
		Fisher	0.2966	0.3182	0.3302	0.338
		MinP	0.0152	0.0042	0.0019	0.0003
	30	CCT	0.0439	0.0577	0.0537	0.0502
		Fisher	0.2964	0.3143	0.3263	0.3339
		MinP	0.0173	0.0059	0.003	0.0002
